# Iterative Joint Estimation Procedure of Channel and PDP for OFDM Systems

**DOI:** 10.3390/e24111664

**Published:** 2022-11-15

**Authors:** Ruixuan He, Xiaoran Liu, Kai Mei, Guangwei Gong, Jun Xiong, Jibo Wei

**Affiliations:** College of Electronic Science and Technology, National University of Defense Technology, Changsha 410073, China

**Keywords:** orthogonal frequency division multiplexing, power-delay profile, linear minimum mean square error, channel estimation

## Abstract

The power-delay profile (PDP) estimation of wireless channels is an important step to generate a channel correlation matrix for channel linear minimum mean square error (LMMSE) estimation. Estimated channel frequency response can be used to obtain time dispersion characteristics that can be exploited by adaptive orthogonal frequency division multiplexing (OFDM) systems. In this paper, a joint estimator for PDP and LMMSE channel estimation is proposed. For LMMSE channel estimation, we apply a candidate set of frequency-domain channel correlation functions (CCF) and select the one that best matches the current channel to construct the channel correlation matrix. The initial candidate set is generated based on the traditional CCF calculation method for different scenarios. Then, the result of channel estimation is used as an input for the PDP estimation whereas the estimated PDP is further used to update the candidate channel correlation matrix. The enhancement of LMMSE channel estimation and PDP estimation can be achieved by the iterative joint estimation procedure. Analysis and simulation results show that in different communication scenarios, the PDP estimation error of the proposed method can approach the Cramér–Rao lower bound (CRLB) after a finite number of iterations. Moreover, the mean square error of channel estimation is close to the performance of accurate PDP-assisted LMMSE.

## 1. Introduction

The OFDM systems based on cyclic prefix (CP) can resist the frequency selective channel and allow for simple one-tap equalization to be performed [1]. However, the performance of the receiver mainly depends on the accuracy of channel estimation. Among the channel estimation methods, least square (LS) estimation is one of the most widely used methods. Although the implementation of LS estimation has low computation complexity, the performance is greatly affected by noise [2]. LMMSE estimation is the theoretically optimal solution when minimizing the MSE. In addition to the noise power, LMMSE also features a channel correlation matrix which depends entirely on channel statistical information. However, in a wide range of systems, the statistical characteristics are unknown at the receiver [3]. Therefore, it is very challenging to achieve high-performance LMMSE estimation.

The channel PDP is often used to describe the statistical characteristics of frequency selective channels. The time dispersion or delay spread of wireless channels is one of the most important parameters in adaptive OFDM systems [4]. For example, the adaptation of OFDM systems such as adaptive modulation and coding, adaptive CP, pilot pattern, and equalization technology [5,6,7] can adjust the transceiver parameters according to the changes of channel time dispersion. These methods enhance system throughput and transmission reliability [8]. When the channel characteristics of time dispersion are available, the inter-symbol interference can be reduced by adjusting the length of the CP [9]. The LMMSE channel estimation also requires the time dispersion information of the channel [10].

The statistics of channel time dispersion include the mean delay spread and the maximum delay spread, which can be estimated by applying cross-correlation of differentially correlated received signals or setting a threshold for the estimated channel impulse response (CIR) [11,12]. However, these condensed parameters may be oversimplified and cannot fully represent the time dispersion characteristics of the channel. To obtain PDP information, the estimation method based on the maximum likelihood criterion of CP is proposed [13]. Although this class of method can obtain better estimation accuracy, the computational complexity is prohibitively high. In addition, the data-assisted methods using a training sequence or pilot can achieve better estimation performance [14]. To solve the problem of generating channel correlation matrix, one approach is to utilize simple PDP models such as the exponential decay or uniform-model. The channel correlation matrix for these PDPs can be simply calculated by estimating the mean delay and the root-mean-squared (RMS) delay spread [15]. However, how to choose an appropriate PDP model is still an open question. The mismatch of a PDP-model and the estimation error of delay parameters will degrade the performance of LMMSE. In response to this problem, a large number of works have proposed to use a pilot to estimate channel PDP, or to achieve LMMSE estimation through approximate PDP [16,17]. Although these methods can effectively avoid the mismatch in frequency domain and the distortion caused by the zero subcarrier, they still cannot guarantee the optimal performance of LMMSE estimation. To solve the problem of PDP model mismatch, the LMMSE estimator with CCF selection function is considered in [18]. The selected CCF is used to construct the channel correlation matrix to avoid the performance loss.

Due to the fact that the PDP is the inverse Fourier transform of the channel frequency correlation, precise channel frequency response can be utilized to estimate exact PDPs. It can be seen that most recent research uses matrix approximation or an inaccurate PDP model to generate channel correlation matrices. Thus, they cannot avoid the performance deterioration of LMMSE. Furthermore, applying independent PDP and LMMSE estimation algorithms is inefficient. This paper proposes an iterative joint estimation procedure for channel LMMSE and PDP. Motivated by [18], the best-matched CCF vector is selected to construct the channel correlation matrix for channel LMMSE estimation. Then, the estimated channel frequency response (CFR) is used for PDP estimation. Iteratively, the estimated PDP can be exploited to update the CCF candidate set. In this way, the estimation performance of both channel and PDP can be improved.

The rest of this paper is organized as follows: Section 2 gives a brief overview of the related work in this field. Section 3 presents the system model and the conventional channel estimation. The proposed algorithm is described in Section 4. Simulations in Section 5 validate the performance of the method. Section 6 concludes this paper and envisages the future work.

Notation: In the following, lower case letter a is used for scalar variable, capital letter A is used for vector variable, the bold letter A is used for matrix, A[m,n] denotes the (m,n)th element of A. ℕ represents the set of natural numbers. ${\left(\cdot \right)}^{T}$, ${\left(\cdot \right)}^{*}$ and ${\left(\cdot \right)}^{-1}$ denote transpose, complex conjugate, and inverse operation, respectively. E{·} represents the expectation operator, |·| and ‖·‖ denotes the modulus of a variable and the Euclidean norm of a vector, respectively.

## 2. Related Work

The methods to obtain the statistical characteristics of time dispersion of channels can be roughly divided into CP-based methods and pilot-based methods. Authors of [19] suggest that the delay and power of multipath components can be determined by the knee-point locations and the gradients of the curve of the CP correlation function. Cui [20] estimates PDP using the maximum likelihood (ML) estimation based on the CP of the OFDM signal. Furthermore, an approximate ML estimation algorithm of PDP and noise variance is proposed in [21] through a simplified joint parameter estimation model. Although the proposed algorithm reduces the complexity of ML estimation, only the suboptimal solution can be obtained. In [13], the LS solution of channel tap-power and its relationship with the correlation coefficient of CP are derived, along with the hypothesis test for identifying the correct path. The proposed method can approach the CRLB of tap-power estimation in quasi-stationary fading channels. However, the existing CP-based methods above may require a large number of OFDM symbols and lead to prohibitively high computational complexity. Instead of using CP, another class of PDP estimation is the data-aided method that exploits the training sequences or pilots. In [22], the level-crossing rate (LCR) in the frequency domain was used for studying the characterization of frequency selectivity channel. It indicates that LCR is proportional to RMS delay spread, and scaling factor depends on the threshold level of observed LCR. However, accurate estimation of LCR requires intensive frequency sampling of the channel response and is highly sensitive to noise, which leads to low spectrum efficiency and reliability. Manhattan distance is used as a symbol-by-symbol correlator to estimate the channel delay parameters in [23]. This method has low computation complexity but limits estimation accuracy. In [24], the Fourier transform of the channel frequency correlation is used to estimate the PDP, and the channel-magnitude-based algorithm is proposed to overcome the sensitivity to timing errors. However, the estimation performance is greatly limited by the accuracy of the channel estimation.

For LMMSE estimation, the channel correlation matrix can be generated by using the simplified PDP model. However, the mismatch of the correlation function will cause estimation performance loss. To reduce the mismatch, Kim and Im [25] suggest using the pilot symbols of all transmit antenna ports to estimate the PDP for multiple-input multiple-output (MIMO) systems. The proposed method alleviates the spectral leakage and the residual noise caused by insufficient samples of the estimated CIR. In [10], the PDP is approximately represented by the curvature of the channel amplitude, and then the Wiener filter coefficients corresponding to the subcarriers are generated by the estimation results. The vector quantization method [26] avoids real-time matrix inversion calculation by calculating the LMMSE filter matrices offline. The filter matrices can be stored in a look-up table and be used for the mapping from the code vector to the quantized LMMSE filter coefficient. To reduce the computational complexity of the channel correlation matrix, discrete cosine transform (DCT)-based methods [27,28] approximate the channel correlation matrix as a diagonal matrix, and the singular value decomposition (SVD) method [29] divides the channel correlation matrix into small submatrix products. However, the approximation in these methods results in performance deterioration. Additionally, Mei [18] proposes to design a candidate set of CCF vectors in advance and then selects the optimal one to construct the channel correlation matrix. This scheme can still achieve good MSE performance when the channel knowledge is completely unknown.

## 3. System Model

The wide-sense stationary-uncorrelated scattering (WSSUS) model is often used to represent time-varying fading channels, in which the instantaneous channel impulse response for baseband can be written by

(1) $$h(t,\tau )=\sum_{l=0}^{L-1}\alpha_{l}(t)\delta \left(\tau -\tau_{l}\right),$$

where L denotes the number of channel paths, $\alpha_{l}(t)$ and $\tau_{l}$ represent the complex amplitude and time delay of the lth multipath component, respectively. For different multipath component l, $\alpha_{l}(t)$ is a wide-sense stationary narrow-band complex Gaussian process that is mutually independent. The mean delay $\bar{\tau }$ and RMS delay spread $\tau_{\mathrm{RMS}}$ of the channel are expressed as

(2) $$\bar{\tau }=\frac{\sum_{l=0}^{L-1}E\left\{{\left|\alpha_{l}\right|}^{2}\right\}\tau_{l}}{\sum_{l=0}^{L-1}E\left\{{\left|\alpha_{l}\right|}^{2}\right\}},$$

(3) $$\tau_{\mathrm{RMS}}=\sqrt{\frac{\sum_{l=0}^{L-1}E\left\{{\left|\alpha_{l}\right|}^{2}\right\}\tau_{l}{}^{2}}{\sum_{l=0}^{L-1}E\left\{{\left|\alpha_{l}\right|}^{2}\right\}}-{\bar{\tau }}^{2}}.$$

By the Fourier transform of the channel impulse response h(t,τ) relative to τ, the channel frequency response at instant t can be obtained as

(4) $$H(t,f)≜\int_{-\infty }^{+\infty }h(t,\tau )e^{-j\pi 2f\tau }d\tau =\sum_{l=0}^{L-1}\alpha_{l}(t)e^{-j\pi 2f\tau_{l}}.$$

Assuming that the number of subcarriers in the OFDM system is N, the CFR of the mth OFDM block and the kth subcarrier in an OFDM data frame are expressed as

(5) $$H[m,k]≜H\left(mT_{b},k\Delta f\right)=\sum_{l=0}^{L-1}\alpha_{l}\left(mT_{b}\right)e^{-j\pi 2k\Delta f\tau_{l}},$$

where k=0,1,⋯,N−1, $T_{b}$ and Δf represent symbol duration and subcarrier spacing, respectively. Using X[m,k] to represent the transmitted data, and removing the CP before the discrete Fourier transform (DFT), the received symbol can be written as

(6) $$Y[m,k]=X[m,k]H[m,k{]e}^{j(2\pi /N)kd_{m}}+W[m,k],$$

where $d_{m}$ is timing error, and W[m,k] is additive white Gaussian noise (AWGN).

Assuming that the number of pilot subcarriers used for estimation is $N_{P}$ and omitting the variable m, the LS channel estimation criterion is given by

(7) $$\begin{array}{ll} {\hat{H}}_{\mathrm{LS}} & =\underset{\hat{H}}{\arg}\min\left\{\left|Y-X\hat{H}\right|\right\}\\ & =X^{-1}Y={\left[\frac{Y[0]}{X[0]}\;\frac{Y[1]}{X[1]}\;\cdots \;\frac{Y[N_{P}-1]}{X[N_{P}-1]}\right]}^{T}. \end{array}$$

LMMSE is the best linear estimation method in terms of MSE performance. Essentially, it involves a weighting matrix Φ to correct the influence of noise on the LS estimation: ${\hat{H}}_{\mathrm{LMMSE}}≜\Phi {\hat{H}}_{\mathrm{LS}}$, where $\Phi =\underset{\Phi }{\arg}\;\min E\left\{{\left\|H-{\hat{H}}_{\mathrm{LMMSE}}\right\|}^{2}\right\}$. Omitting the derivation process, it can be derived as $\Phi =R_{H{\hat{H}}_{\mathrm{LS}}}R_{{\hat{H}}_{\mathrm{LS}}{\hat{H}}_{\mathrm{LS}}}^{-1}$. In order to reduce the number of matrix inversions, the simplified LMMSE channel estimation is given by [30]

(8) $${\hat{H}}_{\mathrm{LMMSE}}=R_{H{\hat{H}}_{\mathrm{LS}}}{\left(R_{HH}+\frac{\beta }{\mathrm{SNR}}I\right)}^{-1}{\hat{H}}_{\mathrm{LS}},$$

where $\beta =E\left\{{\left|x[n]\right|}^{2}\right\}E\left\{{\left|1/x[n]\right|}^{2}\right\},\;n=0,1,\cdots ,N-1$ is a constant and depends on the modulation type. SNR is the average signal-to-noise ratio. $R_{H{\hat{H}}_{\mathrm{LS}}}$ is the cross-correlation matrix of the accurate channel vector and the LS estimation vector, and $R_{HH}$ is the auto-correlation matrix of the channel. The elements in $R_{H{\hat{H}}_{\mathrm{LS}}}$ and $R_{HH}$ satisfy [31]

(9) $$\begin{array}{ll} R_{H{\hat{H}}_{\mathrm{LS}}}[\Delta m,\Delta k] & =R_{HH}[\Delta m,\Delta k]\\ & =E\left\{H[m,k]H^{*}[m+\Delta m,k+\Delta k]\right\}\\ & =R_{H}^{t}[\Delta m]R_{H}^{f}[\Delta k], \end{array}$$

where $R_{H}^{f}$ and $R_{H}^{t}$ are CCF and channel time correlation, respectively. For the same OFDM symbol, the channel time correlation satisfies $R_{H}^{t}[0]=J_{0}(0)=1$, $J_{0}(\cdot )$ is the zeroth-order Bessel function of the first kind. The $R_{H}^{f}$ can be obtained by the Fourier transform of PDP. In addition, if the statistical information of the channel is available, and the PDP decays exponentially, the CCF can be obtained by $R_{H}^{f}[\Delta k]=1/\left(1+j2\pi \tau_{\mathrm{RMS}}\Delta k\Delta f\right)$ [31]. However, the PDP information is usually unknown to the receiver. Therefore, it is challenging to achieve accurate LMMSE estimation.

## 4. Iterative Joint Estimation Algorithm for Channel and PDP

**Figure 1:**
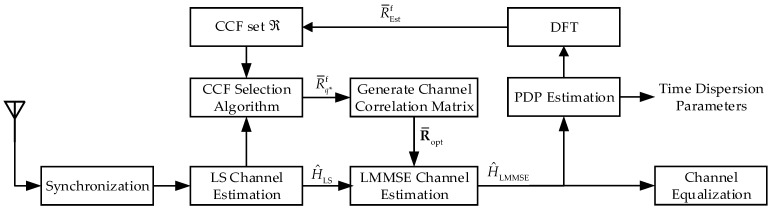
Flow chart of the algorithm at the receiver.

Each frame of the transmitted signal consists of a training sequence and a certain number of data symbols. The CFR of LS estimation ${\hat{H}}_{\mathrm{LS}}$ can be obtained according to (7) by using the training sequence. According to (8), LMMSE estimation needs the channel correlation matrix $R_{HH}$ and SNR. We also assume that the signal power is normalized. Noise power is generally easy to be estimated [32,33]. Therefore, the difficulty of LMMSE estimation mainly depends on the acquisition of the $R_{HH}$. Enabling the estimator with the ability to select CCFs can effectively solve this problem, and we propose a looped algorithmic architecture that enables joint PDP and channel estimation. The execution block diagram of the algorithm at the receiver is shown in Figure 1.

Firstly, we pre-generate a CCF candidate set ℜ, and the CCF vectors are calculated according to different scenarios. Secondly, the CCF selection algorithm is executed to select the best-matched CCF vector ${\bar{R}}_{q*}^{f}$. Then, the ${\bar{R}}_{q*}^{f}$ is used to calculate the channel correlation matrix ${\bar{R}}_{\mathrm{opt}}$ and complete the LMMSE estimation. Further, we use the CFR ${\hat{H}}_{\mathrm{LMMSE}}$ as the input to PDP estimation, and the time dispersion parameters can be estimated. In addition, Fourier transform is applied to the output of PDP estimation, and the result ${\bar{R}}_{\mathrm{Est}}^{f}$ is used to update the CCF set to improve the LMMSE estimation performance. Obviously, the proposed algorithm architecture can be divided into two loops: LMMSE estimation loop and PDP estimation loop. In the following, we describe the specific implementation process of the two loops.

### 4.1. LMMSE Estimation Loop

Let $ℜ=\left\{{\bar{R}}_{1}^{f},{\bar{R}}_{2}^{f},\cdots ,{\bar{R}}_{Q}^{f}\right\}$ be the CCF candidate set, where Q is the cardinality of ℜ and ${\bar{R}}_{q}^{f}={\left[{\bar{R}}_{q}^{f}[0]\;{\bar{R}}_{q}^{f}[1]\;\cdots \;{\bar{R}}_{q}^{f}[N-1]\right]}^{T}$ is the qth candidate CCF vector. When the channel PDP is available, ${\bar{R}}_{}^{f}$ can be easily obtained by Fourier transform of PDP. When the accurate information about the PDP is completely unknown, a robust channel frequency-domain correlation (R-CCF) [34] is required. It can ensure that the loss of channel estimation performance is small, even when there are mismatches between the preset CCFs and the real CCF. A typical R-CCF is the Fourier transform of the uniform spectrum, which can be expressed as

(10) $${\bar{R}}^{f}=\mathrm{DFT}\{[\underset{N_{o}\;\mathrm{elements}}{\underset{︸}{N/N_{o}\;\cdots \;N/N_{o}}}\;0\;\cdots \;0]\},$$

where $N_{o}=\left\lceil \Delta f\tau_{\max}N\right\rceil =\left\lceil \tau_{\max}N/T_{s}\right\rceil$, $T_{s}$, and $\tau_{\max}$ are the system sampling interval (OFDM symbol duration) and the possible maximum delay spread, respectively, and $\tau_{\max}$ usually takes the length of CP.

Moreover, when the timing error is considered, the effective channel frequency-domain correlation function (E-CCF) [31] can be calculated from the typical value of the timing error or the statistical distribution of the timing error. The specific expression can be given as

(11) $$\begin{array}{ll} {\bar{R}}^{f}[\Delta k] & =E\left\{\bar{H}[m,k]{\bar{H}}^{*}[m,k+\Delta k]\right\}\\ & =E\left\{H[m,k{]e}^{j(2\pi /N)kd_{m}}H^{*}[m,k+\Delta k{]e}^{-j(2\pi /N)(k+\Delta k)d_{m}}\right\}\\ & =R^{f}[\Delta k]E\left\{e^{j\pi 2\Delta kd_{m}/N}\right\}\\ & =R^{f}[\Delta k]\sum_{d_{m}=d_{\min}}^{d_{\max}}P(d_{m})e^{j\pi 2\Delta kd_{m}/N}, \end{array}$$

where $P(d_{m})$ is the probability density function of the timing error $d_{m}$. The $R^{f}[\Delta k]$ of (11) can be obtained by the Fourier transform of the PDP. When the channel statistics are completely unknown, the $R^{f}[\Delta k]$ can be substituted by the ${\bar{R}}^{f}$ obtained by (10).

After generating ℜ, the key step is CCF selection. The training sequence is divided into two groups $X_{1}$ and $X_{2}$ at equal intervals in the order of subcarrier index, i.e., $X_{1}=\left[X_{1}[0]\;X_{1}[2]\;\cdots \;X_{1}[N-2]\right]$ and $X_{2}=\left[X_{2}[1]\;X_{2}[3]\;\cdots \;X_{2}[N-1]\right]$. The CFR vectors corresponding to $X_{1}$ and $X_{2}$ are denoted as $H_{1}$ and $H_{2}$, respectively. The corresponding LS estimation vectors are denoted as ${\hat{H}}_{\mathrm{LS}\_1}$ and ${\hat{H}}_{\mathrm{LS}\_2}$, respectively. Then, we use $X_{1}$ to perform MMSE interpolation to estimate $H_{2}$, and use $X_{2}$ to perform MMSE interpolation to estimate $H_{1}$. The interpolation results ${\hat{H}}_{\mathrm{INT}\_1}$ and ${\hat{H}}_{\mathrm{INT}\_2}$ are obtained by

(12) $$\left\{ \begin{array}{l} {\hat{H}}_{\mathrm{INT}\_1}={\bar{R}}_{H_{1}H_{2}}{\left({\bar{R}}_{H_{2}H_{2}}+\frac{\beta }{\mathrm{SNR}}I\right)}^{-1}{\hat{H}}_{\mathrm{LS}\_2}\\ {\hat{H}}_{\mathrm{INT}\_2}={\bar{R}}_{H_{2}H_{1}}{\left({\bar{R}}_{H_{1}H_{1}}+\frac{\beta }{\mathrm{SNR}}I\right)}^{-1}{\hat{H}}_{\mathrm{LS}\_1} \end{array}\right..$$

The evaluation index of the CCF selection algorithm is given by

(13) $$\xi_{n}=\frac{1}{N}\left({\left\|{\hat{H}}_{\mathrm{INT}\_1}-{\hat{H}}_{\mathrm{LS}\_1}\right\|}_{}^{2}+{\left\|{\hat{H}}_{\mathrm{INT}\_2}-{\hat{H}}_{\mathrm{LS}\_2}\right\|}_{}^{2}\right).$$

In Equation (12), $\bar{R}$ is the corresponding channel correlation matrix, whose elements come from the candidate CCF vector ${\bar{R}}_{q}^{f}$. By evaluating each candidate CCF vector according to Equation (13), the CCF that best matches the current channel can be selected as

(14) $$q*=\underset{q}{\arg}\min\xi_{q}.$$

Finally, the CFR of the LMMSE estimator is obtained by

(15) $${\hat{H}}_{\mathrm{LMMSE}}={\bar{R}}_{\mathrm{opt}}{\left({\bar{R}}_{\mathrm{opt}}+\frac{\beta }{\mathrm{SNR}}I\right)}^{-1}{\hat{H}}_{\mathrm{LS}},$$

where ${\bar{R}}_{\mathrm{opt}}$ is the channel correlation matrix constructed by the CCF vector ${\bar{R}}_{q*}^{f}$. It is represented as

(16) $${\bar{R}}_{\mathrm{opt}}≜\left[\begin{array}{cccc} {\bar{R}}_{q*}^{f}[0] & {\bar{R}}_{q*}^{f}[1] & \cdots & {\bar{R}}_{q*}^{f}[N-1]\\ {\bar{R}}_{q*}^{f}[-1] & {\bar{R}}_{q*}^{f}[0] & \cdots & {\bar{R}}_{q*}^{f}[N-2]\\ \vdots & \ddots & \ddots & \vdots \\ {\bar{R}}_{q*}^{f}[-N+1] & {\bar{R}}_{q*}^{f}[-N+2] & \cdots & {\bar{R}}_{q*}^{f}[0] \end{array}\right].$$

Different ℜ may produce different estimation results. Therefore, with updated ℜ, we perform Equations (12)–(15) to complete a new LMMSE estimation loop.

### 4.2. PDP Estimation Loop

The PDP estimation can rely on the principle that the channel frequency correlation and the channel PDP are Fourier transform pairs. The instantaneous channel frequency correlation can be calculated by using the estimated CFR ${\hat{H}}_{\mathrm{LMMSE}}$ as

(17) $$R_{{\hat{H}}_{\mathrm{LMMSE}}}^{f}(\Delta k)=E_{u,k}\left\{{\hat{H}}_{\mathrm{LMMSE},u}[k]{\hat{H}}_{\mathrm{LMMSE},u}^{*}[k+\Delta k]\right\},$$

where u is the index of the frame. The $E_{u,k}\left\{\cdot \right\}$ is the expectation over u and k. The expectation of multiple frames can effectively reduce the impact of noise. Then, the inverse discrete Fourier transform (IDFT) is performed on the obtained channel correlation value to calculate the PDP

(18) $$\begin{array}{ll} P_{l} & =\mathrm{IDFT}\left\{R_{{\hat{H}}_{\mathrm{LMMSE}}}^{f}(\Delta k)\right\}\\ & =\frac{1}{\sqrt{N}}\sum_{\Delta k=0}^{N-1}R_{{\hat{H}}_{\mathrm{LMMSE}}}^{f}(\Delta k)e^{j\pi 2\Delta kl/N},\;0\leq l\leq L-1, \end{array}$$

where $P_{l}=E\left\{{\left|\alpha_{l}\right|}^{2}\right\}\;$ is the power of the lth channel tap. In addition, the taps whose power is less than 25 dB of the strongest tap power are omitted. Then, $\bar{\tau }$ and $\tau_{\mathrm{RMS}}$ are calculated according to Equations (2) and (3), respectively.

Moreover, it is worth mentioning that when using LS-estimated CFR ${\hat{H}}_{\mathrm{LS}}$, the channel frequency correlation obtained according to (16) can be simplified into the sum of effective channel frequency correlation and noise correlation values

(19) $$R_{{\hat{H}}_{\mathrm{LS}}}^{f}(\Delta k)=R_{H}^{f}(\Delta k)+\delta (\Delta k)\sigma_{Z}^{2},$$

where $\sigma_{Z}^{2}$ is the variance of channel estimation error caused by noise, and δ(·) is Dirac delta function. To remove the influence of noise term, we use the correlation value of non-zero lag to fit the parabola by the LS method. Then, replace the correlation value $R_{{\hat{H}}_{\mathrm{LS}}}^{f}(0)$ of zero lag with the polynomial coefficients obtained by fitting. By this means, we can approximate the value of $R_{H}^{f}(\Delta k)$ as ${\tilde{R}}_{H}^{f}(\Delta k)$ and the PDP can be estimated by the inverse Fourier transform of ${\tilde{R}}_{H}^{f}(\Delta k)$.

**Figure 2:**
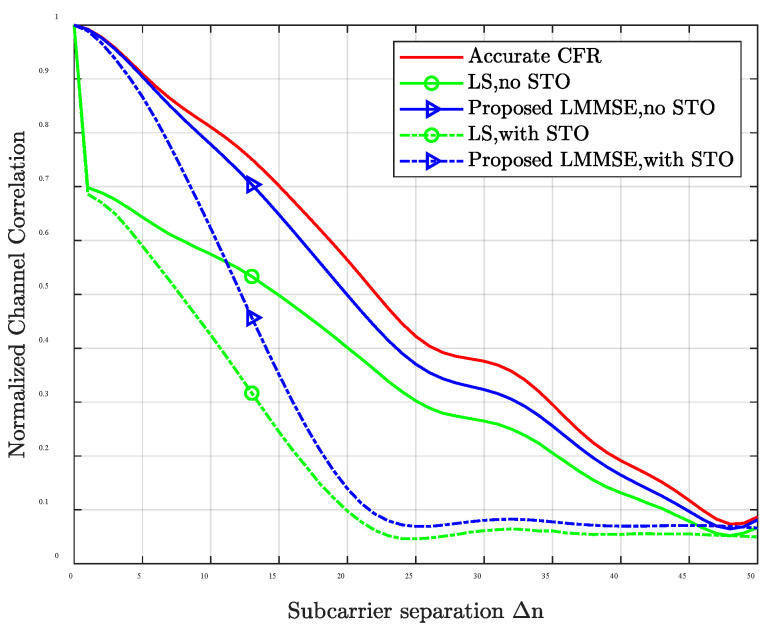
Amplitude of channel frequency correlation with perfect synchronization and considering STO, SNR = 5 dB.

It should be noted that the performance of the proposed PDP estimation algorithm is limited to the accuracy of channel estimation. Figure 2 shows the amplitude curve of normalized channel frequency correlation calculated according to (17) for accurate CFR, LS estimation, and the proposed LMMSE estimation under the ITU-VA channel when the SNR is 5 dB. The dotted lines represent the simulation curves when STO obeys the uniform distribution of [−10, 0]. As can be observed, in the case of perfect synchronization, the channel frequency correlation amplitude of the LMMSE is lower than the accurate CFR but higher than that of the LS channel estimate. In the presence of STO, the channel frequency correlation amplitude of LMMSE and LS shows an obvious reduction. However, the correlation amplitude of LMMSE is still higher than that of LS. That is to say, the proposed LMMSE estimator is able to alleviate the noise effects compared with LS estimation and do not need any prior channel knowledge.

Therefore, we consider using the CFR obtained by the proposed LMMSE estimator as the input of the PDP estimation algorithm instead of the LS estimation value. In addition, with the output of the new LMMSE estimation loop, we can perform a new PDP estimation loop to get a more accurate PDP.

### 4.3. Iterative Joint Estimation Procedure

**Figure 3:**
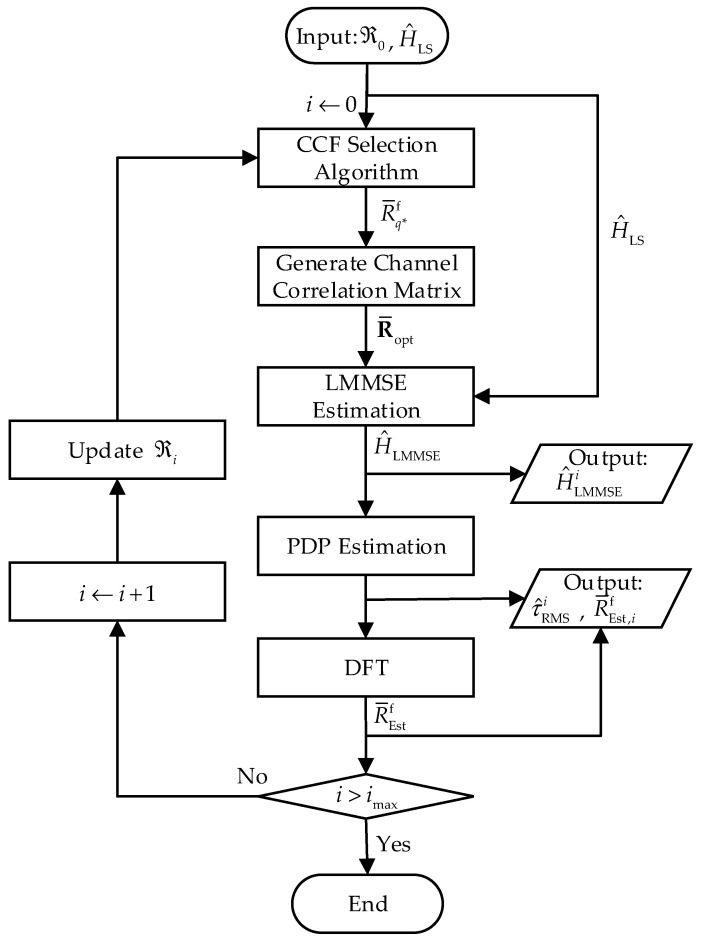
The execution block diagram of the proposed iterative algorithm.

It is evident that the LMMSE estimator can obtain a better CFR in terms of MSE performance than the LS estimator when the channel information is unavailable. Moreover, the performance of the proposed LMMSE estimator depends on the candidate CCF vectors. Thus, we can update the candidate CCF set according to the latest PDP estimation. In this case, the performance of the LMMSE estimation is improved by the updated CCF set. Since the results of each estimation process can be applied to the other one, the proposed algorithm is performed in an iterative manner, as shown in Figure 3.

We denote the number of iterations as i, i∈ℕ and $i_{\max}$ is the maximum number of iterations with i=0 representing the initial process. Firstly, the initial CCF set ${ℜ}_{0}=\left\{{\bar{R}}_{\mathrm{Inl}\_1}^{f},{\bar{R}}_{\mathrm{Inl}\_2}^{f},\cdots ,{\bar{R}}_{\mathrm{Inl}\_Q}^{f}\right\}$ is generated according to (10) and (11) for different scenarios, and the LS estimation value ${\hat{H}}_{\mathrm{LS}}$ is calculated by Equation (7). The ${ℜ}_{0}$ and ${\hat{H}}_{\mathrm{LS}}$ are the inputs of the iterative algorithm. According to the principle of the proposed LMMSE estimator, the CCF selection algorithm is executed to select the CCF vector, ${\bar{R}}_{q*}^{f}$, that best matches the current channel by Equations (12)–(14). Using ${\bar{R}}_{q*}^{f}$ to construct channel correlation matrix ${\bar{R}}_{\mathrm{opt}}$ according to (16), the initial value ${\hat{H}}_{\mathrm{LMMSE}}^{i=0}$ can be obtained by using Equation (15). According to (17), ${\hat{H}}_{\mathrm{LMMSE}}^{i=0}$ is used to calculate the channel frequency correlation, and the results of multiple frames are counted to obtain the expectation. Then, the PDP estimation is completed by Equation (18). On this basis, we can calculate ${\hat{\tau }}_{\mathrm{RMS}}^{i=0}$ according to (3), and a CCF vector ${\bar{R}}_{\mathrm{Est}}^{f}$ can be obtained by performing DFT on the result of PDP estimation. We add i by 1 after each LMMSE estimation and PDP estimation, which means that a new estimation process will begin. Then, we add ${\bar{R}}_{\mathrm{Est},i-1}^{f}$ to the CCF set to obtain a new CCF vector set

(20) $${ℜ}_{i}={ℜ}_{i-1}\cup \left\{{\bar{R}}_{\mathrm{Est},i-1}^{f}\right\}.$$

Based on the above procedure, the channel LMMSE and PDP estimation algorithms are executed iteratively to obtain the channel estimation ${\hat{H}}_{\mathrm{LMMSE}}^{i}$ and the estimated RMS delay ${\hat{\tau }}_{\mathrm{RMS}}^{i}$ of the ith iteration. Because ${ℜ}_{i}$ has more accurate CCF vectors than ${ℜ}_{i-1}$, we can ensure that the performance of each iteration is improved. After each execution of the PDP estimation algorithm, the value of i is judged. The algorithm ends when $i>i_{\max}$. The proposed scheme is summarized as Algorithm 1.
**Algorithm 1:** Iterative joint estimation procedure for channel and PDP**Input**: ${ℜ}_{0}=\left\{{\bar{R}}_{\mathrm{Inl}\_1}^{f},{\bar{R}}_{\mathrm{Inl}\_2}^{f},\cdots ,{\bar{R}}_{\mathrm{Inl}\_Q}^{f}\right\}$, ${\hat{H}}_{\mathrm{LS}}$;**Output**: ${\hat{H}}_{\mathrm{LMMSE}}^{i}$, ${\hat{\tau }}_{\mathrm{RMS}}^{i}$;1. **begin**2.      i←0 
3.      **while** $i\leq i_{\max}$, **do**4.         Execute the CCF selection algorithm (12)–(14)5.         Generate channel correlation matrix ${\bar{R}}_{\mathrm{opt}}$ (16)6.         Perform LMMSE channel estimation and get CFR ${\hat{H}}_{\mathrm{LMMSE}}^{i}$ (15)7.         Using ${\hat{H}}_{E-\mathrm{LMMSE}}^{i}$ to estimate PDP and get the result ${\bar{R}}_{\mathrm{Est},i}^{f}$,${\hat{\tau }}_{\mathrm{RMS}}^{i}$ (17) and (18)8.      **end**9.      i ← i+1
10.      Update the CCF set with ${\bar{R}}_{\mathrm{Est},i-1}^{f}$ to get ${ℜ}_{i}$ (20)11. **end**

### 4.4. Analysis of Computational Complexity

Generally, the LMMSE-based algorithm has significantly higher computational complexity than the one based on the LS estimation proposed in [24]. The process of determining q* needs to calculate the evaluation index $\xi_{q}$ from all candidate CCFs. Therefore, the complexity of the proposed LMMSE estimation loop is proportional to the number of the candidate CCF vectors. Using Equations (12)–(14) to calculate $\xi_{q}$ requires two N/2-order matrix inversion operations. For the candidate CCF set ℜ, we need to calculate Q times of $\xi_{q}$ according to (13), corresponding to 2Q times of N/2-order matrix inversions. When the SNR and q* are determined, the LMMSE estimator needs to calculate ${\bar{R}}_{\mathrm{opt}}{\left({\bar{R}}_{\mathrm{opt}}+(\beta /\mathrm{SNR})I\right)}^{-1}$, once in Equation (15) with $N^{3}$ multiplications and in the N-order matrix inversion. Meanwhile, the LS result ${\hat{H}}_{\mathrm{LS}}$ also needs N times of multiplications. Finally, considering the number of iterations $i_{\max}$, the multiplication and matrix inversion times are counted independently. The results are shown in Table 1, where $L_{r}$ is the length of CP. It can be seen that, compared with the matrix approximation method [27] and the singular value decomposition (SVD) method [29], the LMMSE estimator with CCF selection [18] has a relatively large amount of computation. It is evident that the amount of calculation of algorithm suggested in [24] is minimal since it only performs PDP estimation and needs no matrix inversion operations. Compared with [18], the proposed algorithm only slightly increases the computational complexity when performing both channel LMMSE and PDP estimation.

## 5. Simulation Results and Analysis

In this section, the performance of the proposed iterative joint estimation algorithm is analyzed by simulation. We build an OFDM simulation platform in which the environment parameters are shown in Table 2, and the rest of the system parameters are set according to the IEEE 802.11ax standard. In this paper, we consider using an OFDM symbol-length Zadoff-Chu sequence as the training sequence for channel estimation. The transmitted frame contains 50 data symbols. The results are obtained through 1000 Monte Carlo simulations.

In order to evaluate the performance of channel PDP estimation, we use the normalized mean square error (NMSE) of channel second-order statistics $\tau_{\mathrm{RMS}}^{}$ as the performance metric which can accurately reflect the deviation of the estimator from the theoretical value. The NMSE of $\tau_{\mathrm{RMS}}^{}$ is defined as $\mathrm{NMSE}\left(\tau_{\mathrm{RMS}}\right)≜E\left\{{\left|\tau_{\mathrm{RMS}}-{\hat{\tau }}_{\mathrm{RMS}}\right|}^{2}\right\}/\tau_{\mathrm{RMS}}^{2}$, in which ${\hat{\tau }}_{\mathrm{RMS}}$ represents the estimated values. In addition, the CRLB of the $\tau_{\mathrm{RMS}}^{}$ estimator is used as the comparison [24]

(21) $$\mathrm{CRLB}\left(\tau_{\mathrm{RMS}}\right)=\frac{1}{M_{u}}\sum_{l=0}^{L-1}\frac{P_{i}^{2}}{4\tau_{\mathrm{RMS}}^{2}}{\left(\frac{\tau_{l}^{2}}{\sum_{l}P_{l}}+\frac{\sum_{l}P_{l}\tau_{l}^{2}}{{\left(\sum_{l}P_{l}\right)}^{2}}+\frac{\sum_{l}P_{l}\tau_{l}}{{\left(\sum_{l}P_{l}\right)}^{3}}\left(\tau_{l}\sum_{l}P_{l}-\sum_{l}P_{l}\tau_{l}\right)\right)}^{2},$$

where $M_{u}$ is the number of frames used for estimation. The performance of channel estimation is evaluated by the mean square error (MSE) of CFR: $\mathrm{MSE}(H)=E\left\{{\left\|H-\hat{H}\right\|}^{2}\right\}$ and compared with three typical LMMSE estimation methods [18,27,29].

Since the proposed algorithm is based on the LMMSE estimator, the initial estimation value ${\hat{H}}_{\mathrm{LMMSE}}^{i=0}$ directly affects the performance of the proposed algorithm. The key to the LMMSE estimator lies in the design of candidate set ${ℜ}_{0}$. In order to comprehensively investigate the performance of the proposed algorithm, we divide the possible communication scenarios into three types.

Scenario 1: It is assumed that all possible channel models’ statistics are available. For example, ITU-R defines the four models shown in Table 3. In this scenario, ${ℜ}_{0}$ can be composed of all possible PDPs. In addition, in order to improve the robustness of the estimator, $N_{o}$ takes the value of {4, 16, 64}, and the R-CCFs are also added to ${ℜ}_{0}$ according to (10). The simulated channel of each transmission is randomly selected among four models.

Scenario 2: Consider a more complex communication scenario, where the channel knowledge is unavailable. In this case, ${ℜ}_{0}$ can be obtained by R-CCF and E-CCF. Specifically, three values {4, 16, 64} are set for $N_{o}$, and the corresponding R-CCF is obtained according to (10). In addition, in order to expand the number of CCF, the E-CCF with the value of $d_{m}=[-5,-1]$ is also added to ${ℜ}_{0}$. In the simulation of this scenario, the channel is fixed as the ITU-VA model.

Scenario 3: On the basis of scenario 2, this scenario further considers the non-ideal factor: STO. It is assumed that $d_{m}$ obeys the uniform distribution of [−10, 0]. In this scenario, ${ℜ}_{0}$ and other simulation parameters are the same as in scenario 2.

**Figure 4:**
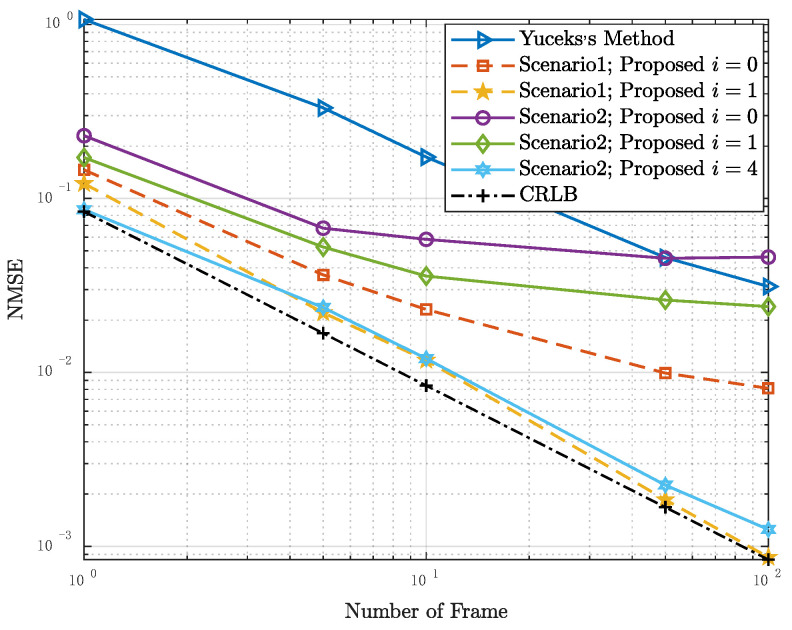
NMSE performance of the RMS delay spread estimators as a function of the number of frames used for estimation in Scenario 1 and Scenario 2, SNR = 5 dB.

Figure 4 shows the relationship between the NMSE of $\tau_{\mathrm{RMS}}$ and the number of frames used to estimate in Scenario 1 and Scenario 2. The label “Yuceks’s Method” represents the algorithm based on the LS estimation proposed in [24]. The “i=0” means the initial result when the algorithm does not start an iteration. That is to say, the candidate CCF set of the LMMSE estimator is ${ℜ}_{0}$. In this case, the estimated result of PDP is calculated with ${\hat{H}}_{\mathrm{LMMSE}}^{i=0}$ as the input. Because the algorithm in [24] is performed based on LS channel estimation, the results are the same in two scenarios, and the MSE are significantly higher than those of the proposed algorithm. The initial result of the proposed algorithm in scenario 1 is better than that in scenario 2. In scenario 1, after one iteration, the NMSE of $\tau_{\mathrm{RMS}}$ is close to CRLB, whereas in scenario 2, it takes four iterations to get a similar result. It is because the ${ℜ}_{0}$ of scenario 1 contains all possible PDPs and it make the algorithm efficient.

**Figure 5:**
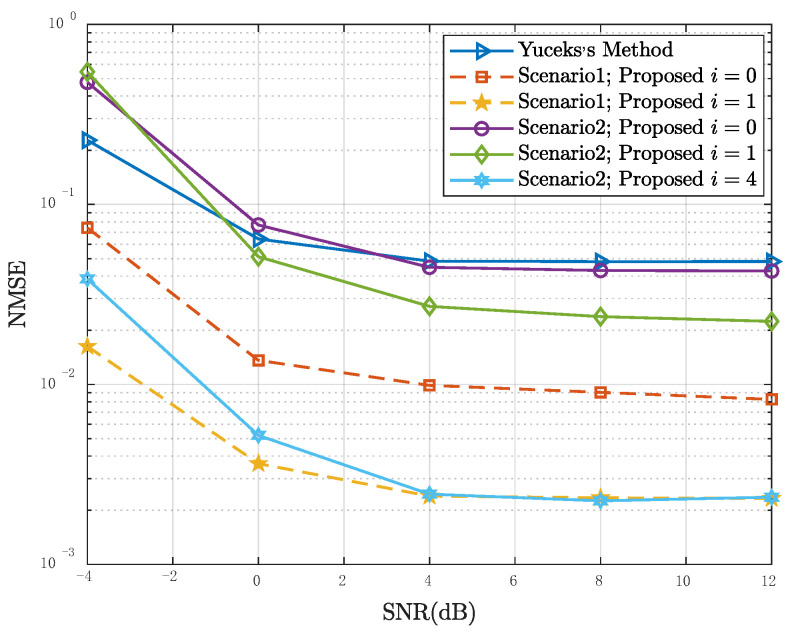
NMSE performance of the RMS delay spread estimators as a function of SNR in Scenario 1 and Scenario 2, $M_{u}$ = 50.

Figure 5 shows the NMSE of $\tau_{\mathrm{RMS}}$ as a function of the SNR in Scenario 1 and Scenario 2, where $M_{u}$ is the number of frames. It can be seen that when the SNR is lower than 4 dB, the performance of the algorithms is improved with the increase of SNR, and the performance of the proposed algorithm is better than the algorithm proposed in [24]. However, when SNR rises to around 5 dB, the NMSE of RMS is SNR-independent. This is due to the limitation of the number of statistical frames.

**Figure 6:**
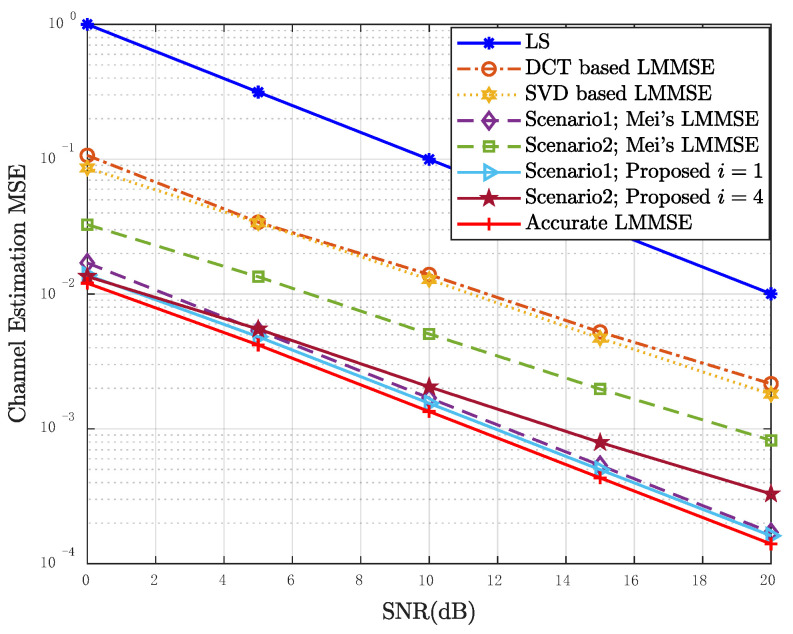
MSE performance of the proposed LMMSE estimator in Scenario 1 and Scenario 2, $M_{u}$ = 50.

Figure 6 simulates the performance of the LMMSE estimation in Scenario 1 and Scenario 2. It should be noted that the results of [24] are also the results of the proposed algorithm when i=0. It can be seen that, compared with the DCT based LMMSE and the SVD based LMMSE in [27,29], the LMMSE with CCF selection is more robust and the proposed iterative algorithm can further improve the performance of LMMSE, especially in scenario 2. The proposed algorithm approaches the accurate LMMSE result after 1 and 4 iterations, respectively, in the two scenarios.

**Figure 7:**
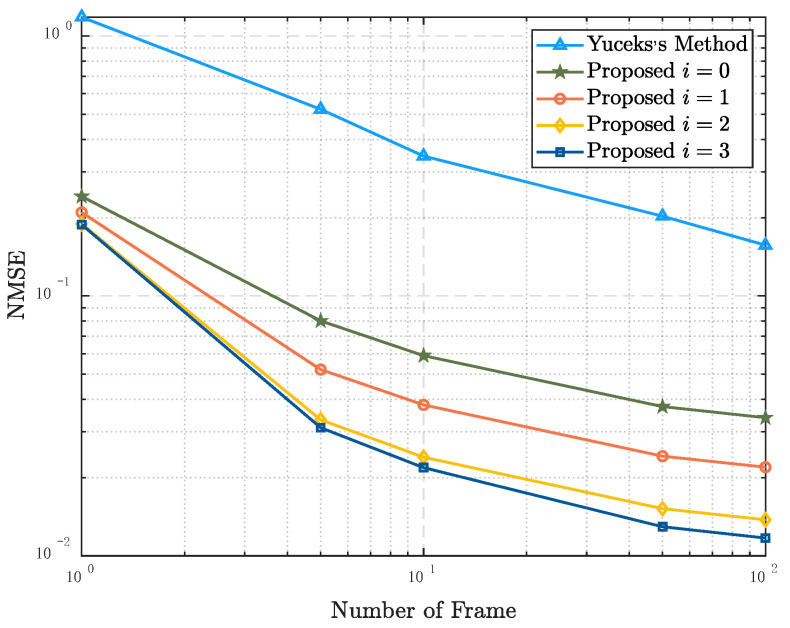
NMSE performance of the RMS delay spread estimators as a function of the number of frames used for estimation in Scenario 3, SNR = 5 dB.

**Figure 8:**
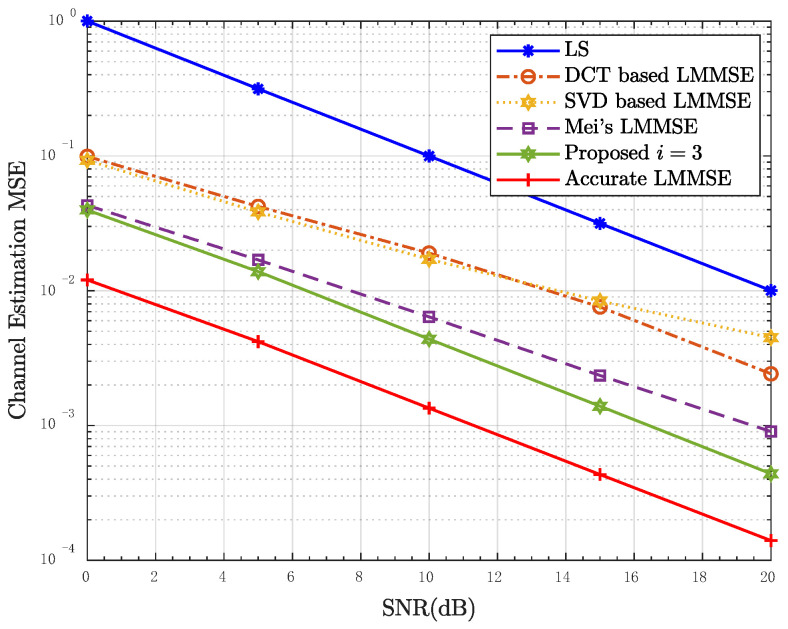
MSE performance of the proposed LMMSE estimator in scenario 3, $M_{u}$ = 50.

Figure 7 and Figure 8 show the simulation results in scenario 3. It can be seen that the performance of PDP estimation is greatly affected by STO, compared with scenarios 1 and 2. The NMSE of $\tau_{\mathrm{RMS}}$ is similar compared to the third iteration and the second iteration. It indicates that the performance of the algorithm cannot be significantly improved after the third iteration. In addition, the performance of the proposed LMMSE estimator still has great advantages over the other three algorithms in this scenario.

## 6. Conclusions and Future Work

In this paper, we propose a joint estimation algorithm for channel and PDP, including the LMMSE estimate loop and PDP estimate loop. The proposed LMMSE estimator has the ability to select the best-matched CCF, which solves the problem of channel correlation matrix generation. The CFR obtained is used for PDP estimation, and the results can be applied to update the CCF set. Since both PDP and channel estimation requires the result from the other one, an iterative joint algorithm is proposed. The proposed algorithm is evaluated by the NMSE of the second-order statistic of PDP and the channel MSE. Simulation results show that the performance of PDP estimation is better than that of existing LS-based methods in different scenarios. The NMSE of RMS can approach CRLB after a limited number of iterations. The results of this paper can be used to obtain accurate PDP results and improve the performance of channel estimators in OFDM systems. As for future extensions of this work, we will consider further performance improvement by the joint estimation process of channel response and Doppler spread. By generating the candidate set of channel correlation function and choosing the best-matched correlation function vector, the channel frequency response estimated by LMSME can be used to estimate the Doppler spread. Therefore, the estimated results can update the channel correlation matrix. A joint estimation process of channel and the Doppler spectrum can also be obtained. This approach is appealing especially in adaptive OFDM systems.

## Figures and Tables

**Table 1:** Computational complexity comparison.

	Multiplication	N-Order Matrix Inversion	N/2-Order Matrix Inversion
Yuceks’s method [24]	$N^{2}+N/2\log_{2}N$	—	—
DCT based LMMSE [27]	$2N^{2}$	1	—
SVD based LMMSE [29]	$(L_{r}+2)N^{2}+L_{r}N$	—	—
Mei’s LMMSE [18]	$(Q/4+1)N^{3}+(Q/2+1)N^{2}$	1	2Q
Proposed iterative joint estimation algorithm	$\begin{array}{c} \;(i_{\max}+1)((Q/4+1)N^{3}\\ +(Q/2+1)N^{2})+N^{2}+N/2\log_{2}N \end{array}$	$i_{\max}+1$	$\left(i_{\max}+1)(i_{\max}+2Q\right)$

**Table 2:** Simulation parameter settings.

Simulation Parameters	Value
Bandwidth	10 MHz
Center frequency	2.4 GHz
Modulation type	16 QAM
FFT size	256
CP length	64
Channel model	ITU-R

**Table 3:** PDPs of ITU-R model.

Model	Parameter	Path Number					
		1	2	3	4	5	6
Pedestrian A	Delay (ns)	0	110	190	410	—	—
	Power (dB)	0	−9.7	−19.2	−22.8	—	—
Pedestrian B	Delay (ns)	0	200	800	1200	2300	3700
	Power (dB)	0	−0.9	−4.9	−8.0	−7.8	−23.9
Vehicle A	Delay (ns)	0	310	710	1090	1730	2510
	Power (dB)	0	−1.0	−9.0	−10.0	−15	−20.0
Vehicle B	Delay (ns)	0	300	8900	12,900	17,100	20,000
	Power (dB)	−2.5	0	−12.8	−10.0	−25.2	−16.0

## Data Availability

Not applicable.
